# Prevalence and associated factors of uncorrected presbyopia among adults in cross-sectional Fujian Eye Study

**DOI:** 10.3389/fmed.2026.1745527

**Published:** 2026-02-27

**Authors:** Yang Li, Qinrui Hu, Bin Wang, Xiangdong Luo, Mingqin Zhang, Shengqi Su, Xiaoxin Li

**Affiliations:** 1 Eye Institute and Affiliated Xiamen Eye Center of Xiamen University, School of Medicine, Xiamen University, Xiamen, China; 2 Xiamen Clinical Research Center for Eye Diseases, Xiamen, Fujian, China; 3 Xiamen Key Laboratory of Ophthalmology, Xiamen, Fujian, China; 4 Translational Medicine Institute of Xiamen Eye Center of Xiamen University, Xiamen, Fujian, China; 5 Fujian Provincial Key Laboratory of Corneal & Ocular Surface Diseases, Xiamen, Fujian, China; 6 Xiamen Municipal Key Laboratory of Corneal & Ocular Surface Diseases, Xiamen, Fujian, China; 7 Xiamen Science and Technology Middle School, Xiamen, China; 8 Department of Ophthalmology, Peking University People's Hospital, Beijing, China

**Keywords:** associated factor, cross-sectional study, elderly population, prevalence, uncorrected presbyopia

## Abstract

**Objective:**

With the improvement of people's living standards, the demand for eye health is no longer limited to being visible, but more importantly, being able to see clearly. The aging population has led to increasing attention on presbyopia worldwide. Our study aims to reveal the prevalence and related factors of uncorrected presbyopia among urban and rural adults over 50 years of Fujian Province Southeast China.

**Methods:**

A population-based cross-sectional study was performed to evaluate the prevalence and related factors of presbyopia among urban and rural adults over 50 years in more than 50 communities of Fujian Province Southeast China from May 2018 to October 2019. A total of 8,211 residents aged over 50 years underwent a questionnaire and a series related examinations. Presenting near visual acuity (PNVA) was measured using logarithmic visual acuity chart at a distance of 40 cm, and uncorrected presbyopia was defined as PNVA worse than N6.

**Results:**

Among the 8,033 residents included in this study, the prevalence of uncorrected presbyopia was 68.6% (*n* = 5,509). Multivariable regression analysis identified older age, less educated, lower income, higher intraocular pressure (IOP), and higher spherical equivalent (SE) as factors independently associated with uncorrected presbyopia. In contrast, sex, height, weight, systolic blood pressure (SBP), outdoor time, phone usage (including use in the dark), history of hypertension, history of hyperlipidemia, smoking, alcohol consumption, tea consumption, and degree of urbanization showed no independent association after adjustment.

**Conclusions:**

There was a significant burden of uncorrected presbyopia in southeast China, which suggested more investment in accessible services and policy to enhance eye health especially on less educated and low income elderly with high IOP and refractive error. Although age is a significant correlate, its value in discriminating presbyopia in an individual is modest at best within this age-homogeneous population.

## Introduction

Presbyopia is an age-related near vision impairment (NVI) that manifests early as “aging-induced lens optical dysfunction” or dysfunctional syndrome. Proper presbyopia treatment depends on coexisting factors, such as reduced contrast sensitivity, increased higher-order spherical aberrations, light scattering and lens opacity (1). Therefore, presbyopia will seriously affect the quality of life of individuals during the life course. With the increase in people's average age, the modern working age has also increased. Presbyopia has seriously affected the productivity of working-age people, which has a significant impact on economic development. Actually, the leading cause of NVI, an age-related public health concern, is uncorrected presbyopia throughout the world, which can be corrected with a pair of spectacles (2, 3). There are 1.8 billion people (95% confidence interval (CI): 1.7–2.0 billion) suffering from presbyopia globally, as per global estimates. Of these, 826 million had near vision impairment because they had no or inadequate vision correction (4). Functional presbyopia affected 80% of patients aged 50 years or older until 2015. In 2020, 82.2% of people aged 50 years and over had VI from uncorrected presbyopia globally. It has been predicted that by 2050, 866 million (95% CI: 629–1,150 million) people will have uncorrected presbyopia (5). Because of unmet needs and an aging global population, eye health is a major public health and sustainable development concern that warrants urgent political action (6).

Recently, the prevalence and associations of presbyopia have attracted increasing attention worldwide. However, presbyopia was only reported based on robust data from several countries, and high-quality prevalence data were available for only 4 countries (7–10). There are few studies focusing on presbyopia in China as well (11, 12). In regard to comparison, there is still a need to increase consistency in the methodology of presbyopia studies (13). In addition, most of the studies reported the correlation of presbyopia with urbanization separately (7, 11), and there was no correlation analysis on geographic location. Therefore, our study primarily aimed to assess the prevalence of presbyopia among residents aged 50 years and over in Fujian Province, southeast China. We also aimed to study the associations of uncorrected presbyopia with various sociodemographic factors, including both urbanization and geographic location. Moreover, our research was based on the same baseline data to increase the credibility for comparison, which could provide the latest and most credible data to support policy-making efforts.

## Materials and methods

### Study design

The Fujian Eye Study (FJES) is a population-based, cross-sectional survey on the public eye health status of residents aged 50 years and above in Fujian Province, southeast China. This study adopted random cluster sampling design. The following formula was used to calculate the required sample size: *n* = (deff) × μα^2^ × *p* × (1 – *p*)/(*d*^2^). For this study, a precision of 0.05 (*d*) is aimed for, with a 95% confidence interval (two-tailed), where μα^2^ is 1.96, the design effect is 2, and the relative error is 0.15. The precision, *d*, is equal to *r* × *p*. The sample size is intended to ensure sufficient precision in estimating prevalence rates and to conduct risk factor analyses. Given that the prevalence of major eye diseases in cross-sectional baseline surveys is estimated to be more than 2.0% (14–17), and considering the response rate from the pilot study (approximately 85%), a total of 10,044 participants are to be enrolled in this study. The proportion of the survey population is based on data from the National Bureau of Statistics for the year 2017 (18).

### Patient and public involvement statement

The sampling strategy for the Fujian Eye Study employed a two-stage, stratified, cluster random sampling design to ensure a representative sample of adults aged 50 years and above across Fujian Province (19).

First Stage: Selection of Primary Sampling Units (Clusters/Communities).

The sampling frame was stratified by the nine prefecture-level cities within Fujian Province. Within each city stratum, communities (clusters) were selected with probability proportional to size (PPS). The “size” measure was the number of officially registered residents aged ≥50 years in each community, based on local government registries. This PPS method ensured that larger communities (with more eligible residents) had a proportionally higher chance of being selected, enhancing the efficiency and representativeness of the sample at the provincial level.

Second Stage: Selection of Individuals within Clusters.

Within each selected community (cluster), a pre-determined number of participants were randomly sampled from the community registry. The target sample size for each city was calculated based on its proportion of the provincial population aged ≥50 years (according to 2017 National Bureau of Statistics data). This city-level sample size was then allocated proportionally to its selected communities. Therefore, the final selection of individuals was also effectively probability proportional to the size of their community within the sample allocation.

Justification and Weighting Consideration: this design aimed to generate a self-weighted or approximately self-weighted sample for estimating population-level prevalence within the surveyed age group. While complex sampling effects were accounted for in variance estimation during analysis (e.g., using cluster-robust standard errors), post-stratification or design weights were not applied for the primary descriptive and regression analyses presented, as the core objective was to assess associations within the sampled population.

### Ethics approval and consent to participate statement

A clinical study registry was obtained for the 2018–2019 FJES study (Register number: ChiCTR2100043349, registration date: 2021-02-21) and the Human Ethics and Consent to Participate declarations was approved by the Ethics Committee of Xiamen University Xiamen Eye Center (Acceptance number: XMYKZX-KY-2018-001), and written informed consent was obtained from all participants.

### On-site examination

The main contents of this report include the following: the questionnaire includes sections on basic information, overall health and medication history, eye conditions, and lifestyle habits, among other survey content, such as age, sex, and race; educational background; income level; occupation; height, weight, systolic blood pressure (SBP), diastolic blood pressure (DBP), history of hypertension (HT), diabetes mellitus (DM), or hyperlipidemia (HL); smoking, drinking, and tea drinking history, which was developed by our own team and was provided as attachment named the English Version of Questionnaire; intraocular pressure (IOP), presenting near visual acuity (PNVA) and spherical equivalent refraction (SE).

Refractive state [spherical (DS), cylinder (DC), axis (a)] were conducted three times by Topcon KR800 (Topcon, Japan) in each eye, and a mean value was calculated as the final result.

NVA was measured by logarithmic visual acuity chart at a distance of 40 cm. And uncorrected presbyopia was defined as PNVA worse than N6 (20) (equaled with 0.5 in our logarithmic near visual acuity chart). Participants were instructed to perform the test using their habitual, daily-life visual condition. This means: participants who normally used distance spectacles (e.g., for myopia) were not asked to remove them. Their NVA was tested with their habitual distance correction in place. Participants were not provided with any near-vision optical aids (e.g., reading glasses) for the test, as the aim was to assess their functional near vision in a typical, uncorrected state for near tasks. Testing was performed monocularly, following the standard protocol for visual acuity assessment in large-scale epidemiological studies. And the data from the right eye were used for analysis in this study, consistent with common practice.

An experienced technician measured participants' IOP with the handheld iCare rebound tonometer (RBT; Icare TA 01, Finland). Quintic measurements were obtained, measurements with poor signal quality (indicated by the device) were automatically discarded and not included in the set of five valid readings required for the average and their mean was recorded and taken for further statistical analysis. The RBT has demonstrated good operator internal repeatability in field studies. Perform by the same operator to improve reliability. In randomized controlled and observational studies, the iCare rebound tonometer has been validated with the Goldmann flattening tonometer, showing good correlation and reproducibility.

Smoking/Drinking/Tea Drinking: these variables were defined as “current habitual use” (yes/no), based on self-report.

Phone Usage was a binary (yes/no) self-report of current smartphone use. “Phone usage time” was categorized based on self-reported average daily use: <1 h, 1–2 h, >2 h.

The variable ‘phone use in dark environments' was based on participant self-report in our questionnaire: ‘Do you use mobile phone after turning off the lights at night?' Responses were recorded as a binary variable (Yes/No). This measure was intended to capture a common behavioral pattern rather than a precisely quantified photometric state.

Outdoor Time: This was categorized based on self-reported average daily time spent outdoors on weekdays: <2 h, 2–4 h, >4 h.

### Data collation and statistical analysis

Double entry with EpiData v3.1 (EpiData for Windows, version 3.1, the EpiData Association, Denmark, Europe) was used for data collation. Each resident has a unique identification code to accurately locate their data. We supplemented part of the missing data through telephone follow-up. If we can't contact the resident, it is considered as missing data.

Data analysis was carried out using Stata/SE statistical software (Stata for Windows, version 15.1, StataCorp LLC, Lakeway Drive, College Station, TX, USA). Prevalence of presbyopia was calculated and presented with 95% confidence intervals (CIs). Chi-square (χ^2^) tests were employed for analyzing categorical variables. Linear regression analysis was used to examine the relationship between uncorrected presbyopia and potential risk factors. Logistic regression was utilized to assess the degree of correlation within each group. Multivariable logistic regression was performed to evaluate the association of various factors with uncorrected presbyopia. The regression analyses were performed using complete-case analysis, the final analytic sample size will be specified based on the variables included. Multicollinearity among independent variables in the multivariable model was assessed using variance inflation factors (VIF). Odds ratios (ORs) or correlation coefficients (r values) along with their 95% CIs were reported. A *P*-value of less than 0.05 was deemed statistically significant for all the estimates.

## Results

### Characteristics of the participants

From an initial enumeration of 8,211 residents aged 50 years and above, 8,033 were included in the final analysis following the exclusion of those without presenting near visual acuity (PNVA) data. The prevalence of uncorrected presbyopia in this cohort was 68.6% (*n* = 5,509). This subgroup with uncorrected presbyopia was characterized as follows: The mean age was 65.34 ± 0.12 years (95% CI: 65.11–65.56), 62.2% female (*n* = 3,428), 55.4% from urban areas (*n* = 3,057), 78.0% from coastal regions (*n* = 4,300), 86.0% with any formal education (*n* = 4,735), and 58.9% with any level of income (*n* = 3,244) (Table 1).

**Table 1:** The characteristics of the study participants and the prevalence of uncorrected presbyopia (n = 8033)

**Characteristics**		**Total population**	**Presbyopia**	***P* value**	***P*-Holm**
Age (years, mean ± SD)		64.35 ± 0.10	65.34 ± 0.12	<0.001	<0.001
Intraocular pressure (IOP, mmHg, mean ± SD)		13.87 ± 0.04	13.81 ± 0.05	0.019	0.114
Sex	Male	3197	2081 (65.1%)	<0.001	<0.001
	Female	4836	3428 (70.9%)		
Urbanization	Urban	4554	3057 (67.1%)	0.001	0.005
	Rural	3479	2452 (70.5%)		
Geographic location	Coastal	6299	4300 (68.3%)	0.247	n.s.
	Inland	1734	1209 (69.7%)		
Education level	Illiteracy	1272	1049 (82.5%)	<0.001	<0.001
	Primary school	1500	1186 (79.1%)		
	Middle school	3049	1974 (64.7%)		
	College and above	1162	526 (45.3%)		
	Missing data	1050	774 (73.7%%)		
Income level	≤ 2000	2392	1830 (76.5%)	<0.001	<0.001
	2000–5000	1914	1144 (59.8%)		
	>5000	572	270 (47.2%)		
	Missing data	3155	2265(71.8%)		
Spherical equivalent (SE, Diopters) group	SE <−10.00	97	60 (61.9%)	<0.001	<0.001
	−10.00 ≤ SE <−6.00	99	52 (52.5%)		
	−6.00 ≤ SE <−3.00	282	103 (36.5%)		
	−3.00 ≤ SE <0.00	1148	428 (37.3%)		
	SE = 0	320	143 (44.7%)		
	0.00 <SE ≤ +3.00	5360	4106 (76.6%)		
	+3.00 <SE ≤ +5.00	312	273 (87.5%)		
	+5.00 <SE ≤ +10.00	41	36 (87.8%)		
	SE > +10.00	4	3 (75.0%)		
	Missing data	370	305(82.4%)		
Total		8033	5509 (68.6%)		

*P*-values are presented for descriptive statistics and univariable comparison. Given the multiple comparisons, significance should be interpreted with caution. The primary inference for associations is derived from the regression models presented in Tables 2 and 3.

*P*-Holm values exceeding 1 after Holm correction were reported as n.s.

### Subgroup analysis and distribution of uncorrected presbyopia

**Figure 1:**
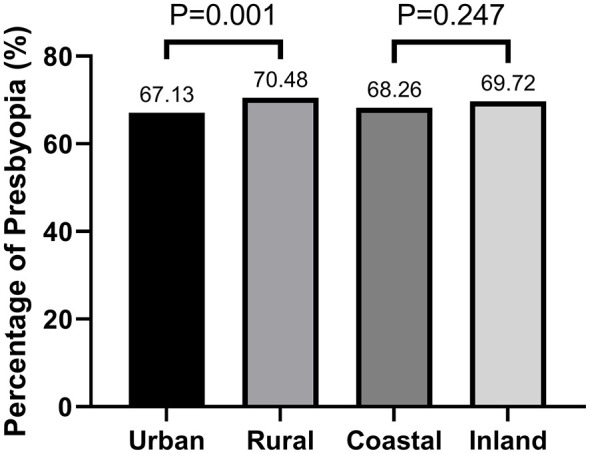
The comparison of the prevalence of presbyopia among different regional subgroups.

In the whole study population, There is statistical difference in the prevalence of uncorrected presbyopia (*P* = 0.001) between urban and rural areas. The percentage of uncorrected presbyopia was significantly lower in the urban group than that in the rural group. While there is no difference (*P* = 0.247) in geographical location (Table 1 and Figure 1).

**Figure 2:**
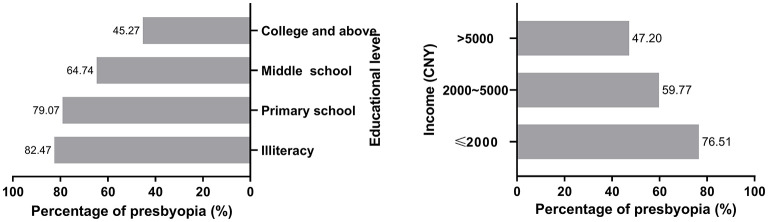
The comparison of the prevalence of presbyopia among education and income subgroups.

Given that the urban population group and inland population group had a significantly (*P* < 0.001) higher level of education, all populations were stratified into subgroups based on the level of education (*P* < 0.001, Table 1). With the improvement of education level, the prevalence of uncorrected presbyopia gradually decreased (Figure 2). Income groups showed the similar results (*P* < 0.001, Table 1). As the income level increased, the prevalence of uncorrected presbyopia also gradually decreased (Figure 2).

**Figure 3:**
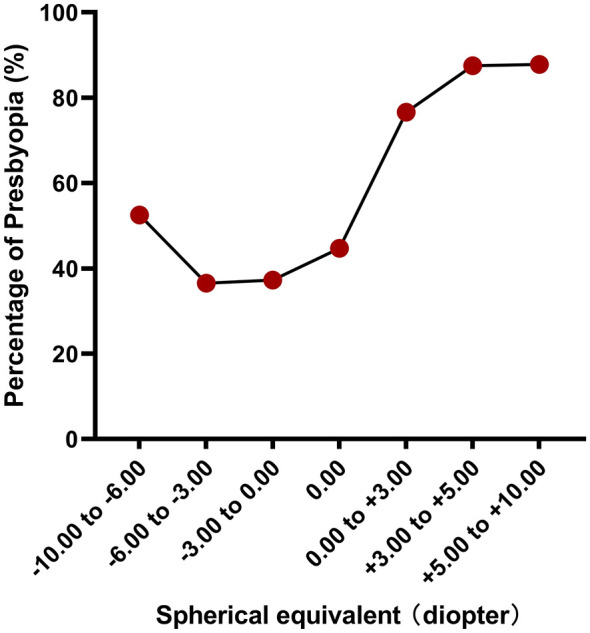
The comparison of the prevalence of presbyopia among Spherical equvalent subgroups.

There is also a statistically significant difference in the prevalence of uncorrected presbyopia among different refraction groups (*P* < 0.001, Table 1). All populations were also stratified for subgroups according to the refractive error level. With the exception of the extreme groups, the prevalence of uncorrected presbyopia among the mild to middle myopia groups was the lowest. Moreover, with the deviation of diopters, presbyopia increased significantly (Figure 3).

### Correlation of uncorrected presbyopia with social-demographic and biological factors

In general, uncorrected presbyopia was significantly correlated with lower urbanization (OR = 0.855, *P* = 0.001), older age (OR = 1.043, *P* < 0.001), female sex (OR = 1.306; *P* < 0.001), lower height (OR = 0.976, *P* < 0.001), lower weight (OR = 0.988, *P* < 0.001), higher SBP (OR = 1.006, *P* < 0.001), history of HT (OR = 1.255, *P* < 0.001), none history of HL (OR = 0.733, *P* < 0.001), no or less phone use (OR = 0.405 and 0.667, *P* < 0.001), no phone use in the dark (OR = 0.531, *P* < 0.001), less outdoor activity time (OR = 0.822, *P* = 0.003), smoking (OR = 0.822, *P* = 0.003), alcohol consumption (OR = 0.601, *P* < 0.001) and tea consumption (OR = 0.666, *P* < 0.001), less educated (OR = 0.541, *P* < 0.001), lower income (OR = 0.505, *P* < 0.001), lower IOP (OR = 0.984, *P* = 0.019) and higher SE (OR = 1.785, *P* < 0.001), while it was independent with geographic location (OR = 0.934, *P* = 0.247), DBP (OR = 0.998, *P* = 0.233) and history of DM (OR = 1.050, *P* = 0.364). Detailed results are shown in Table 2.

**Table 2:** The univariable logistic regression of several social-demographic and biological factors on uncorrected presbyopia in Fujian Eye Study (n = 8033).

**Characteristics**	**Odds Ratio**	***p* value**	**95% Confidence Interval**
Urbanization	0.855	0.001	0.777–0.941
Geographic location	0.934	0.247	0.832–1.048
Sex	1.306	<0.001	1.187–1.437
Age	1.043	<0.001	1.037–1.049
Educational background	0.541	<0.001	0.511–0.574
Income level	0.505	<0.001	0.463–0.552
Refractive error (Diopters) group	1.785	<0.001	1.707–1.867
Height	0.976	<0.001	0.970–0.982
Weight	0.988	<0.001	0.984–0.993
Systolic blood pressure	1.006	<0.001	1.004–1.009
Diastolic blood pressure	0.998	0.233	0.994–1.001
Intraocular pressure	0.984	0.019	0.970–0.997
History of Hypertension	1.255	<0.001	1.142–1.379
History of Hyperlipidemia	0.733	<0.001	0.644–0.833
History of Diabetes Mellitus	1.050	0.364	0.946–1.165
Phone usage or not	0.405	<0.001	0.359–0.457
Phone usage time group	0.667	<0.001	0.634–0.701
Phone usage in the dark or not	0.531	<0.001	0.477–0.592
Outdoor activity time group	0.899	0.003	0.840–0.964
Smoking	0.822	0.003	0.722–0.937
Drinking	0.601	<0.001	0.537–0.686
Tea drinking	0.666	<0.001	0.599–0.741

### Multiple logistic regression

A multiple logistic regression analysis was performed. The parameters of age, sex, degree of urbanization (urban and rural), height, weight, SBP, IOP, SE, educational background, income, outdoor time, phone usage or not, phone usage in the dark or not, history of HT, history of HL, smoking or not, drinking or not and tea drinking or not were all analyzed, and associations with uncorrected presbyopia was showed in Table 3. The multiple logistic regression showed that uncorrected presbyopia was correlated with older age, lower income, less educated, higher IOP and higher SE, while sex, height, weight, SBP, outdoor time, phone usage or not, phone usage in the dark or not, history of HT, history of HL, smoking, drinking, tea drinking and the degree of urbanization were no longer correlated with uncorrected presbyopia. The VIF results were showed in Table 4 and the mean VIF values was 1.77, indicating no severe multicollinearity.

**Table 3:** The multiple logistic regression of several social-demographic and biological factors on uncorrected presbyopia in Fujian Eye Study.

**Research factors**	**Uncorrected presbyopia**		**95% confidence interval**
	**Odds Ratio**	***P*** **-value**	
Urbanization	1.021	0.831	0.840–1.242
Sex	1.027	0.849	0.780–1.354
Age	1.148	<0.001	1.079–1.222
Height	1.008	0.324	0.992–1.025
Weight	0.994	0.270	0.982–1.005
Systolic blood pressure	1.003	0.313	0.997–1.009
Intraocular pressure	1.030	0.031	1.003–1.058
Refraction group	1.665	<0.001	1.538–1.803
History of hypertension	0.955	0.712	0.750–1.217
History of hyperlipidemia	0.807	0.063	0.644–1.012
Education	0.716	<0.001	0.627–0.817
Income	0.776	0.002	0.661–0.910
Outdoor time group	0.955	0.383	0.861–1.059
Phone usage or not	0.766	0.098	0.558–1.051
Phone usage time group	1.052	0.433	0.927–1.194
Phone usage in the dark or not	0.868	0.169	0.710–1.062
Smoking	1.014	0.924	0.771–1.333
Drinking	0.817	0.101	0.641–1.040
Tea drinking	0.980	0.845	0.804–1.196

**Table 4:** Variance inflation factors for variables included in the multivariable logistic regression model.

**Variable**	**Variance inflation factors (VIF)**
Age	1.36
Sex	2.31
Urbanization	1.12
Height	2.21
Weight	1.67
Systolic blood pressure	1.9
Intraocular pressure	1.06
History of hypertension	1.87
History of hyperlipidemia	1.07
Outdoor time group	1.06
Phone usage or not	2.44
Phone usage in the dark or not	1.27
Phone usage time group	2.41
Spherical equivalent group	1.07
Smoking	1.6
Drinking	1.33
Tea drinking	1.25
**Education groups**	
2	2.13
3	3.14
4	3.41
**Income groups**	
2	1.49
3	1.72
Mean VIF	1.77

## Discussion

With the increase in aging and the changes in modern lifestyle, more elderly people who originally had a more basic lifestyle have increased their use of smartphones. NVI, especially presbyopia, is one of the most important factors affecting vision function and quality of life among elderly individuals. A multinational study highlighted that the impact of nearsightedness on vision function should receive greater emphasis with further investigation in various socioeconomic and cultural settings (21). Therefore, in-depth research on presbyopia and how to more accurately address the eye-health requirements of different groups of elderly individuals is imminent. Although there have been a large number of studies on presbyopia in the past, recent large-sample studies in the coastal province of southern China are still lacking. This study included data from 2019, which can be used as baseline data before the epidemic and provide a basis for future research.

### Prevalence of presbyopia

The reported presbyopia prevalence varied across regions and by age group (7–12, 22–28). The prevalence of uncorrected presbyopia in our study was 68.6%. The multiple logistic regression showed that presbyopia was correlated with older age, lower income, less educated and higher refractive error, while sex, smoking, alcohol consumption, tea consumption, geographic location and the degree of urbanization were no longer correlated with presbyopia. Lancet reported that functional presbyopia affected an estimated 666·7 million people (80% UI 364·9-997·6) aged 50 years or older until 2015. In 2020, the population aged 50 years and over accounted for an estimated 419 million (82.2%, 295–562) of 510 million (371–667) people with VI from uncorrected presbyopia globally. By 2050, they predict that 866 million (629–1,150) people will have uncorrected presbyopia (5). In a study of a population aged 45 years and older from urban and rural areas of Parintins in the Brazilian Amazon region, presbyopia was the principal cause of NVI in 71.8% of the participants (22). The prevalence of unmanaged presbyopia is as high as 50% of those over 50 years of age in developing world populations due to a lack of awareness and accessibility to affordable treatment and is even as high as 34% in developed countries (23). The study among subjects aged ≥35 years in Yuexiu District, Guangzhou, China showed the prevalence of functional presbyopia at baseline was 25.2% (11). At present, there is still a lack of standard epidemiological survey norms for comparison of global big data. The large difference in the prevalence of different research findings may be related to many factors, such as different definitions, countries, regions, age groups, and proportions of different populations. Therefore, epidemiological studies should also formulate a unified standard for comparison of the prevalence.

### Correlation on presbyopia

This study showed that uncorrected presbyopia was correlated with older age, lower educational background, and lower income and refractive error, independent of sex, degree of urbanization, geographic location, smoking, alcohol consumption and tea consumption. There have also been many reports on the analysis of factors related to presbyopia worldwide (4, 9, 22, 24). For example, in a multiethnic Asian population and the Brazilian Amazon Region, younger age was associated with higher odds of uncorrected presbyopia (*P* < 0.05) (22, 24). The Andhra Pradesh Eye Disease Study among people aged 30 years and older also reported that female sex, rural residence, myopia, and hyperopia were associated with presbyopia (9). A global systematic review reported that people with presbyopia were more likely to have adequate optical correction if they lived in an urban area of a more developed country with higher health expenditure and lower inequality (4), potentially because participants in our study discounted the importance of near vision correction and the gap between urban and rural areas in our region was not very obvious. These differences may be because our research results were finally obtained by multivariable regression analysis, excluding the influence of some confounding factors. Our study population was 8,033 individuals aged 50 years and older, while the Brazilian population was 2025 individuals aged 45 years and older, and other studies also included different ages and regions.

These studies have only reported correlations between certain factors and presbyopia (11, 12, 22, 24, 25). A multinational study highlighted that the impact of nearsightedness on vision function should receive greater emphasis with further investigation in various socioeconomic and cultural settings (21). According to our refractive subgroup, uncorrected presbyopia in the mild to moderate myopia population was relatively low, whereas that in the high myopia population was high. With the increase in diopter, the uncorrected presbyopia prevalence of the hyperopia population also gradually increased. With the increase in myopia, uncorrected presbyopia gradually increased synergistically, but the prevalence was still lower than that of hyperopia overall. Hyperopia is still the group with the highest degree of uncorrected presbyopia. This result was consistent with previous studies worldwide (4, 9, 22, 24) and was consistent with the development trend of ocular refraction. Our results also showed that with an increase in education and income level, uncorrected presbyopia significantly decreased. A systematic review reported that the greatest burden of vision impairment caused by uncorrected presbyopia was in rural areas of low-resource countries (4). In a multiethnic Asian population, lower education and income levels were associated with higher odds of uncorrected presbyopia (all *P* < 0.05) (24), which was also consistent with our study. However, the result was inconsistent with the study from the Brazilian Amazon Region, which showed that presbyopia was associated with high-school education status (22). A study by Nirmalan et al. and a seven-site multicountry study found no correlation between education and presbyopia (9, 26). Our study demonstrated that more attention needs to be paid to populations with low-to-middle education and income level, and those populations need more convenient policies to be developed. Other studies also showed that people with less education were at greater risk of uncorrected presbyopia, possibly because those with lower educational levels were likely to be of lower socioeconomic status and therefore have fewer resources to care for their eyes (27, 28). At the same time, the lower the educational background, the poorer the awareness of vision care. The insights from the multi-ethnic Asian study conducted by Kidd Man et al. among subjects aged 40–86 years in Singapore indicated that, within their multivariable model, younger age, male sex, Malay and Indian ethnicity, the presence of hyperopia (in either eye), and lower education and income levels were all associated with higher odds of uncorrected presbyopia (all *P* < 0.05) (24). While some of these findings align with conclusions from our study, notable discrepancies also exist. These differences may highlight the potential influence of ethnic background—and by extension, variations in ocular development or biological predisposition—on the epidemiology and presentation of presbyopia.

A novel and intriguing finding of our study is the identification of a statistically significant, albeit marginal, positive association between lower intraocular pressure (IOP) and uncorrected presbyopia in the univariable model (OR = 0.984, *P* = 0.019), while positive association between higher intraocular pressure (IOP) and uncorrected presbyopia in the multivariable model (OR=1.030, *P* =0.031). Although statistically significant, the small effect size suggests a limited direct clinical or etiological relevance for IOP as a standalone risk factor for uncorrected presbyopia. The association represents an odds ratio per 1 mmHg increase in IOP. The reversal of direction between models strongly suggests confounding, most likely by age and spherical equivalent, and potential influences from the complete-case analysis. Therefore, the small, positive association should not be interpreted as evidence of a clinical or etiological risk factor for presbyopia. Its clinical relevance is minimal, and it is best understood as a statistical artifact within the specific model. This association may be interpreted through several non-causal, age-related physiological pathways. First, it may reflect shared anatomical changes associated with aging. Age-related thickening and increased rigidity of the crystalline lens (29, 30)—the primary pathogenic basis of presbyopia—can alter anterior segment biomechanics and potentially influence IOP measurement dynamics. Second, a slight elevation in IOP could be a secondary marker of reduced aqueous outflow facility, which itself is associated with aging and may co-vary with other lenticular changes (31). Importantly, this finding should not be conflated with pathological ocular hypertension. Recent investigations into the biomechanics of accommodation and presbyopia have highlighted the complex interplay between ciliary body function, zonular tension, lens properties, and anterior segment parameters, which may indirectly involve IOP as a correlated variable within this system (32). For instance, a 2024 study by Fan et al. showed that drinking cassiae tea improves dry eye symptoms, reduces IOP, regulates pupil size, and enhances near vision due to its excellent antioxidant and pharmacological properties (33). However, our observation likely captures a weak statistical linkage within the multivariable model rather than evidence of a causative role. This underscores the importance of interpreting such marginal associations with caution. Future longitudinal studies employing detailed anterior segment imaging (e.g., AS-OCT) and dynamic IOP measurements are warranted to explore whether any true biomechanical link exists or if this association is merely an epiphenomenon of co-occurring age-related ocular changes.

No differences in uncorrected presbyopia were revealed between coastal and inland regions in our study, and this result was reported for the first time worldwide. Another study showed that presbyopia was associated with residing in remote geographical areas in both indigenous Australians aged 40 years and over and nonindigenous Australians aged 50 years and over (34). This may suggest that geographic location may not be an important associated factor with uncorrected presbyopia in Fujian Province, southeast China, and that near vision correction requires attention. Additionally, no statistically significant difference was noted in people with uncorrected presbyopia between urban and rural areas, which differed from the results of several other studies. A study in northern Iran (35) and the Brazilian Amazon Region Eye Study (22) showed that presbyopia prevalence in urban areas was lower than that in rural areas, potentially because participants in our study discounted the importance of near vision correction.

### Interpretation of multivariable analysis findings

A notable finding of this study was the attenuation of statistical significance for several factors—including sex, degree of urbanization, and lifestyle habits such as smoking, alcohol, and tea consumption—when moving from univariable to multivariable logistic regression models. This shift can likely be attributed to confounding effects and structural overlap among variables. Socioeconomic factors, particularly education and income, emerged as robust, independent correlates of uncorrected presbyopia in our final model. These variables are closely intertwined with other demographic and lifestyle characteristics. For instance, urban residence is often associated with higher educational attainment and income, which may facilitate greater access to vision care services and awareness. When education and income are accounted for statistically, the independent contribution of urbanization *per se* diminishes. Furthermore, the age-homogeneous nature of our population (≥50 years) likely accentuates the dominant role of age as a covariate, which may further attenuate the independent contribution of other variables. This pattern suggests that in presbyopia epidemiology, socioeconomic determinants (education, income) and core biometric factors (age, refractive error) may constitute more primary pathways influencing both its prevalence and correction, overshadowing the direct effects of certain demographic or behavioral variables in a multivariable context. We acknowledge that this pattern differs from some prior studies where, for example, sex or rural-urban disparities remained independently significant. Future longitudinal studies are warranted to disentangle these complex relationships and establish causal pathways.

### Strengths

Large and well-defined sample: this study was based on a substantial sample size of 8,033 adults from the Fujian Eye Study, enhancing the statistical power and generalizability of the findings within the southeastern Chinese population.

Comprehensive risk factor analysis: we systematically investigated a wide array of potential associated factors, including demographic, socioeconomic, lifestyle, and ocular parameters, providing a holistic view of presbyopia correlates in this region.

Robust statistical methodology: the use of multivariable regression analysis helped to identify independent associated factors by controlling for potential confounders.

Novel and policy-relevant findings: this study is among the first to report no significant association between uncorrected presbyopia and geographic location (coastal vs. inland) or degree of urbanization in this context. The observation of the prevalence trend stabilizing after the general retirement age provides unique, policy-oriented insights for workforce and public health planning.

### Limitations

Firstly, the inherent nature of a cross-sectional study prevents the establishment of causal relationships between the identified factors and presbyopia.

Secondly, a primary limitation was the uncorrected presbyopia assessment based on uncorrected near visual acuity alone without measuring best-corrected near visual acuity (BCNVA). Consequently, the study reports the prevalence of uncorrected presbyopia and could not analyze severity grades of presbyopia, which may limit direct comparisons with studies using different definitions. Thirdly, a significant methodological constraint was our inability to account for cataract status and cataract surgery history, including the type of intraocular lens (IOL) implanted. These data were not systematically collected in the baseline survey of the Fujian Eye Study. This omission represents a notable source of potential confounding and limits the interpretability of our findings in several key aspects. Crucially, both cataract opacity and cataract surgery with modern IOLs can profoundly influence near visual performance at a 40 cm testing distance. For example, multifocal, extended depth-of-focus (EDOF), or monovision IOL strategies are explicitly designed to improve uncorrected near and intermediate vision (36–38). Therefore, our primary outcome—presenting near visual acuity—could reflect not only the physiological decline of accommodation (presbyopia) but also the mixed effects of lens pathology and surgical correction. This confounding likely biases the observed age-prevalence relationship. The apparent stabilization or slight decrease in uncorrected presbyopia prevalence in the oldest age groups (e.g., 70–79 years) may partly be attributable to higher rates of cataract surgery with presbyopia-correcting IOLs in these cohorts, rather than indicating a true biological plateau. Consequently, the age trends presented in this study should be interpreted with caution. Furthermore, access to cataract surgery and premium IOLs is often correlated with socioeconomic factors such as income and education, which were independent correlates in our model. The lack of data on surgical status means we could not control for this pathway, potentially altering the strength or even the direction of the observed associations for socioeconomic variables. Future longitudinal studies on presbyopia must prioritize the comprehensive collection of lens status and IOL design data to disentangle these complex interactions.

## Conclusion

Above all, this study identifies that older age, lower educational and income levels, higher IOP, and higher SE are associated with uncorrected presbyopia among residents aged over 50 years in Fujian Province of southeast China. Sex, height, weight, SBP, DBP, degree of urbanization, geographic location, outdoor time, phone usage or not, phone usage in the dark or not, history of hypertension, history of hyperlipidemia, smoking, drinking and tea drinking were independent of uncorrected presbyopia. This study provided the latest uncorrected presbyopia data before the epidemic, which played a theoretical and practical supporting role in future policymaking and may be useful for monitoring and planning primary eye care services. Since the cause of presbyopia is complicated, our observations could be incomplete, and more in-depth explorations are still needed.

## Data Availability

The original contributions presented in the study are included in the article/Supplementary material, further inquiries can be directed to the corresponding author.
